# Identification and Antioxidant Activity of the Extracts of *Eugenia uniflora* Leaves. Characterization of the Anti-Inflammatory Properties of Aqueous Extract on Diabetes Expression in an Experimental Model of Spontaneous Type 1 Diabetes (NOD Mice)

**DOI:** 10.3390/antiox4040662

**Published:** 2015-10-09

**Authors:** Nayara Simon Gonzalez Schumacher, Talita Cristina Colomeu, Daniella de Figueiredo, Virginia de Campos Carvalho, Cinthia Baú Betim Cazarin, Marcelo Alexandre Prado, Laura Maria Molina Meletti, Ricardo de Lima Zollner

**Affiliations:** 1 Laboratory of Translational Immunology, Faculty of Medical Sciences, University of Campinas, Rua Vital Brazil, 300, Cidade Universitária Zeferino Vaz, 13083-888 Campinas-SP, Brazil; E-Mails: nayaraschumacher@yahoo.com.br (N.S.G.S.); talitacolomeu@yahoo.com.br (T.C.C.); daniellafig@gmail.com (D.F.); vi.carvalho28@hotmail.com(V.C.C.); 2 Department of Food and Nutrition, School of Food Engineering, University of Campinas (FEA-Unicamp), Rua Monteiro Lobato, 80, Cidade Universitária Zeferino Vaz, 13083-862 Campinas-SP, Brazil; E-Mails: cinthiabetim@gmail.com (C.B.B.C.); marcelo.prado@reitoria.unicamp.br (M.A.P.); 3 Agronomic Institute of Campinas, Theodureto de Almeida Camargo, 1500, Vila Nova, 13012-970 Campinas-SP, Brazil; E-Mail: lmmm@iac.sp.gov.br; 4 Department of Internal Medicine, Faculty of Medical Sciences, University of Campinas, Rua Tessália Vieira de Camargo, 126, Cidade Universitária Zeferino Vaz, 13083-887 Campinas-SP, Brazil

**Keywords:** *Eugenia uniflora*, type 1 diabetes mellitus, NOD mice, antioxidant

## Abstract

Medical and folklore reports suggest that *Eugenia uniflora* (*E. uniflora)* is a functional food that contains numerous compounds in its composition, with anti-inflammatory, antioxidant and anti-diabetic effects. In the present study, we investigated the best solvents (water, ethanol and methanol/acetone) for extracting bioactive compounds of *E. uniflora* leaves, assessing total phenols and the antioxidant activity of the extracts by 2,2-Diphenyl-1-picrylhydrazyl (DPPH), Ferric Reducing Antioxidant Power (FRAP), 2,2′-Azinobis (3-ethylbenzothiazoline-6-sulfonic acid) (ABTS) and Oxygen Radical Absorbance Capacity (ORAC) assays, identifying hydrolysable tannins and three phenolic compounds (ellagic acid, gallic acid and rutin) present in the leaves. In addition, we evaluated the incidence of diabetes, degree of insulitis, serum insulin, hepatic glutathione and tolerance test glucose in non-obese diabetic (NOD) mice. Our results suggest that the aqueous extract presents antioxidant activity and high total phenols, which were used as a type 1 diabetes mellitus (DM-1) treatment in NOD mice. We verified that the chronic consumption of aqueous extract reduces the inflammatory infiltrate index in pancreatic islets, maintaining serum insulin levels and hepatic glutathione, and reducing serum lipid peroxidation as well as the risk for diabetes.

## 1. Introduction

The *Eugenia* L. genus belongs to the family Myrtaceae and has more than 500 species, of which 400 are native to Brazil and are used as medicinal plants. *Eugenia uniflora* (*E. uniflora*), popularly known as Surinam cherry, belongs to this genus and is found in regions with tropical and subtropical climates, where it is prized for its fruit [1]. Due to the therapeutic activities of *E. uniflora* tea, made from its leaves, this plant has been studied for its effectiveness in treating various diseases and its application in folk medicine as an antioxidant, hypotensive, anti-inflammatory and hypoglycemic agent [2].

The main mechanism of action of antioxidants include radical scavengers and suppressors that neutralize or eliminate reactive oxygen species (ROS)/nitrogen (RNS) and the binding of metal ions, which are necessary for the production of oxidizing species [3]. Antioxidants may be classified as endogenous (glutathione peroxidase, catalase and superoxide dismutase) and also as exogenous from our diet, such as Vitamins A, C and E, minerals and flavonoids, among others [4]. The presence of these endogenous or exogenous components may be essential for the complex control of oxidative stress and cell damage. Phenolic compounds derived from plant sources are widely studied antioxidant compounds and act in the neutralization of free radicals, helping to control the oxidative stress that occurs in the pancreatic islets of diabetic rats [4]. These compounds are divided into: phenolic acids, phenylpropanoids, flavonoids, condensed and hydrolyzed tannins [5,6]. Metabolic diseases, such as diabetes mellitus, are characterized by a decrease in endogenous antioxidants in the body, leading to an increase in free radicals. Thus, dietary supplementation with antioxidant compounds (present in *E. uniflora* leaves) in diabetes may constitute a support strategy for diabetes treatment.

NOD mice are used as an experimental DM-1 model because they spontaneously develop a similar disease to that observed in humans. Diabetes onset in NOD mice starts between the 12th and 24th weeks of life. Polydipsia, polyuria, high glycosuria, hyperglycemia and insulin deficiency are observed in these animals, accompanied by a rapid loss of weight [7,8]. Among mechanisms proposed to break immune tolerance in DM-1, the genetic predisposition of an individual, together with environmental factors such as stress and diet, seems to contribute to the inflammatory autoimmune response. As such, researchers continue to search for potential new drugs present in medicinal plants. Thus, the aim of the present work was the identification of phenolic compounds with antioxidant and anti-inflammatory properties in *E. uniflora* leaf extract, with a view to their use in the treatment of inflammatory diseases, such as type 1 diabetes mellitus.

## 2. Experimental Section

### 2.1. Extraction of Phenolic Compounds in the Eugenia uniflora Leaves

Samples selected to compose this project are not on the list of endangered species, according to IBAMA-CITES (Convention on International Trade in Endangered Species of Wild Fauna and Flora). The identification of samples was performed by the Agronomic Institute of Campinas. 

*E. uniflora* leaves were dried in a circulating air oven at 50 °C/48 h, ground and stored under refrigeration at 8 °C, and then submitted to a process for extracting phenolic compounds, using three different solvents at the same concentration (0.04 mg/mL), *i.e*., water, ethanol and methanol/acetone. The aqueous extract was prepared using 1 g of sample and 25 mL of water in an autoclave system for 20 min/121 °C, according to our previous study [9]. The tea made by the process of autoclaving was filtered and stored at 8 °C. The ethanol extract was adapted from Spagolla *et al.* (2009) [10]. One gram of sample was suspended in 15 mL of 60% ethanol and maintained for 1 h at 70 °C. The sample was filtered, the supernatant suspended in 10 mL of 60% ethanol in the residue and the material was extracted again for 1 hour. The sample was stored at 8 °C. The preparation of methanol/acetone extract was adapted from Larrauri *et al*. (1997) [11]. One gram of sample was suspended in 10 mL of 50% methanol at room temperature (24 °C), for 60 min. The sample was then centrifuged at 9948× *g* for 20 min/22 °C. The supernatant was added to 10 mL of 70% acetone for another 60 min, at room temperature, and the sample was centrifuged again. The supernatants of the three prepared extracts were filtered and stored at 8 °C for further chemical analysis.

### 2.2. Chemical Analysis

#### 2.2.1. Determination of Antioxidant Activity of Extracts

Extracts (aqueous, ethanol and methanol/acetone) of *E. uniflora* were submitted to four different assays for antioxidant activity analysis at an initial concentration of 0.04 g/mL. DPPH radical scavenging [12]; capacity to reduce metal iron FRAP [13,14]; inhibition of free radical ABTS [11,15] and by peroxyl radical capture ORAC [16].

#### 2.2.2. Determination of Total Phenolic Compounds

Total phenolic compounds in the extracts were determined by the Folin-Ciocalteu method with some modifications [17]. Gallic acid was used to construct the standard curve for phenol analysis, varying the concentrations from 0.016 to 0.1 mg/mL. The absorbance was read using a spectrophotometer (Spectramax 190, Molecular Devices, Sunnyvale, CA, USA) at 725 nm, and the results were expressed as gallic acid equivalents (GAE mg/g sample).

#### 2.2.3. Identification of Hydrolysable Tannins

The qualitative analysis of tannin was determined by Viana *et al.* (2012) [18]; 2 mL of each extract were added in 5 mL of distilled water and 100 μL of 2% ferric chloride. The presence of tannins was determined by the precipitate color and formation, where green precipitates indicate condensed tannins and blue hydrolysable tannins.

The quantification of hydrolysable tannins was determined according to Brune *et al*. (1992) [19] with some modifications by Schons and Macedo *et al.* (2011) [20], using the ferric ammonium reagent FAS-reagent. The standard curve was performed using tannic acid (Sigma, Aldrich, St. Louis, MO, USA) at concentrations between 0.01 and 0.4 mg/mL; after 15 min, the absorbance of the reaction product was read using a spectrophotometer (Spectramax 190) at 578 nm. Results were expressed as tannic acid equivalent (mg of tannic acid/g sample).

### 2.3. Chromatographic Analysis (HPLC)

Validation methodology employed the external standards of the Food Analysis Laboratory, FEA-Unicamp. The extracts were diluted in 80% methanol and additional identification of antioxidant flavonoids-gallic acid, vanillic acid, ellagic acid, *p*-coumaric acid, ferulic acid, kaempferol, resveratrol, quercetin, catechin, epicatechin and rutin, were performed using the standard stock solution (10 mg/mL HPLC grade) to obtain a 5-point calibration curve. The HPLC system used was HPLC (Agilent Technologies 1100, Santa Clara, CA, USA), coupled to a diode array detector (DAD) (Agilent G1315B) with a flow rate of 0.70 mL/min, at room temperature. The chromatographic column used in the reversed-phase was the Eclipse XBB-C18 (Agilent Technologies). Detection was performed at 210, 254, 300 and 340 nm for the identification of phenolic compounds. The mobile phase consisted of 1% orthophosphoric acid in ultrapure water (A) and 100% acetonitrile (B). The elution gradient used was as follows: 0 min: 95% A and 5% B, 10 min: 75% A and 25% B, 25 min: 60% A and 40% B, followed by a linear increase of solvent A until 35 min. Phenolic compounds were identified in the chromatograms of the injected samples and compared with the phenolic standards used, with regard to relative retention time (RT), peak area percentage and spectral data.

### 2.4. Biological Assays: NOD Mice Treatment with E. uniflora Leaves

#### 2.4.1. Experimental Design

Female NOD mice used in this study were obtained from the Multidisciplinary Center for Biological Research, University of Campinas (Cemib-Unicamp) [21]. Procedures involving animals and their care were conducted in accordance with guidelines and recommendations established by the Brazilian Committee for Animal Experimentation—COBEA (protocol: 2824-1).

All animals were treated from the 4th until the 26th week of life, and body weight and fasting blood glucose levels were monitored weekly, beginning at 10th week of life until the end of the protocol, using glucometer strips (Medisense Optium^®^, Abbott Diabetes Care Inc., Alameda, CA, USA).

The experimental group consisted of 57 mice fed on a chow diet (Labina-Purina, Paulínia, Brazil) kept under specific pathogen free (SPF) conditions, with controlled light, temperature and humidity in the Laboratory of Translational Immunology (LTI) facilities. The groups were divided into: Group composed of 27 NOD mice treated with autoclaved aqueous extract of *E. uniflora* leaves *ad libitum* (0.06 g/100mL of filtered water standards established by the IC50-DPPH analysis). Based on previous trials of antioxidant activity and microbiological control, it was established that the aqueous extract of exchanges would offer animals every two days. For each new leaf extract, the DPPH technique was performed to determine the concentration to be used. The second experimental group was classified as untreated, and composed of 30 NOD mice. Thus, from these groups, a third subgroup was designated as the acute diabetic group, and composed of untreated and treated mice that became diabetic during the time of protocol study. In addition to the experimental design, another 36 NOD female and 12 BALB/c mice (healthy control mice that do not develop diabetes) [22] were used for glucose tolerance test.

#### 2.4.2. Diagnosis of Acute Diabetes and Animal Sacrifice

At the end of 26 weeks or mice diabetes onset (blood glucose above 250 mg/dL for two consecutive days), the animals were sacrificed by intraperitoneal anesthesia (ketamine hydrochloride: 150mg/kg and xylazine hydrochloride: 10mg/kg, both Vetbrands, Paulínia, Brazil) and peripheral blood was collected by cardiac puncture for serum separation. Liver was removed and homogenized in 5% trichloroacetic acid (TCA) and frozen at −80°C to further analysis of reduced glutathione (GSH). After this procedure, the pancreas was removed and included into capsules containing tissue freezing medium (Triangle Biomedical Sciences, Durham, NC, USA), frozen in liquid nitrogen and stored at −80 °C for histological analysis.

#### 2.4.3. Histological Analysis

The fixed pancreas were submitted to cryosections (Cryostat CM 1850, Leica Biosystems, Wetzlar, Germany) for morphological analysis of pancreatic islets and insulitis degree classification (inflammatory cell infiltration). A series of 15 consecutive cuts of 5 μm were placed on silanized slides (Methacryl-oxypropyl-Methoxysilane, Sigma). The slide numbers 1, 15, 16, 30, 31 and 45 were stained using hematoxylin and eosin technique (HE) and analyzed by optical microscopy (Nikon^®^ Eclipse 80i, Tokyo, Japan) to count pancreatic islets and for classification of the insulitis.

**Figure 1:**
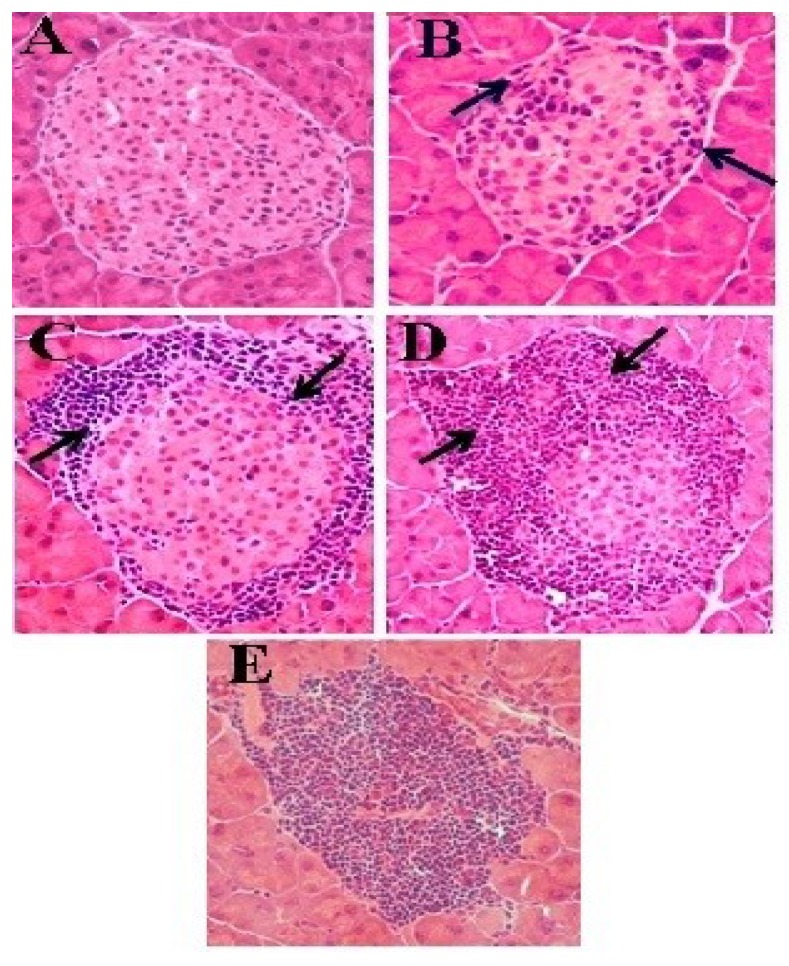
Representative image of pancreatic islets of NOD mice at different grades of cell infiltration. (**A**) Grade 0 (0%); (**B**) Grade 1 (<25%); (**C**) Grade 2 (25%–80%); (**D**) Grade 3 (>80%) and (**E**) Grade 4 (100%).

The analysis of the islets was carried out according to the criteria established by Signori *et al*. (1989) [23], and adapted by Ventura *et al.* [24], where grade 0 is characterized by absence of inflammatory cell infiltrate, grade 1 was less than 25%, grade 2 when the islet has a cell infiltration of between 25% and 80%, grade 3 represents higher than 80% and less than 100%, while islets completely overtaken by cellular infiltration are classified as islets grade 4 (Figure 1). The insulitis index was determined according to Leiter (2001) [25], considering 300 islets/group (5 mice/group).

#### 2.4.4. Detection of Serum Insulin Levels

Serum insulin was determined by rat/mouse insulin ELISA kit, according to the instructions of the manufacturer (Millipore^®^, Billerica, MA, USA). After four hours of fasting, mice were sacrificed and their whole blood removed by cardiac puncture and serum separation. Absorbance was read on a spectrophotometer (Spectramax 190) at 450 nm and 590 nm, and the curve drawn using four parameters analysis.

#### 2.4.5. Glucose Tolerance Test

The glucose tolerance test (GTT) was performed according to Andrikopoulos *et al*. (2008) [26], with some modifications. Mice (15–28 weeks old) were divided into the following groups: NOD mice treated for one month with *E. uniflora* aqueous extract (*n* = 12), untreated NOD mice (*n* = 12), acute diabetic NOD mice (*n* = 12) and BALB/c (healthy control mice), which do not develop diabetes (*n* = 12). The animals were fasted for 4 hours, followed by dosing of glucose at time 0 (blood sample from the caudal vein) using measuring strips (Medisense Optium^®^, Abbott Diabetes Care Inc. Alameda, CA, USA). After measuring at time 0, 25% glucose was administered by intraperitoneal injection (1 g/kg/weight), and blood samples were collected at 10, 30, 60, 90 and 120 min followed by the glucose quantified at each time.

#### 2.4.6. Reduced Glutathione Analysis

Protein concentration and reduced glutathione (GSH) of liver homogenates were determined according to the Hartree (1972) [27] and Faure and Lafond (1995) [28], respectively. Standard curves (2.5–500 nmol/mL), 100 μL of Tris/EDTA buffer (1mM Tris/2 mM EDTA) and 20 μL of reactive DTNB 10 mM (5,5′-Dithiobis(2-nitrobenzoic acid) were used in GSH analysis. Absorbance was read at 412 nm in a spectrophotometer (Spectramax 190). The calculation of glutathione was made by determining the glutathione/total protein ratio and the result was expressed as nmol GSH/mg protein.

#### 2.4.7. Determination of Serum Antioxidant Activity and Lipid Peroxidation Assay

Serum antioxidant activity in NOD mice was determined by the inhibition of free radical ABTS and the results were expressed as μM trolox/g sample [11,15].

The concentration of lipid peroxidation product or thiobarbituric acid reactive substances (TBARS) was determined in the serum of NOD mice, according to Ohkawa *et al*. (1979) [29] with modifications [30]. The samples were mixed with 8.1% sodium dodecyl sulphate (SDS) plus working reagent (TBA, 20% acetic acid, and 5% sodium hydroxide). After heating at 95°C for 60 min, the samples were maintained in an ice-bath for 10 min, and centrifuged at 10,000 g for 10 min. The supernatant was read at 532 nm in Synergy HT, Biotek microplate reader (Winooski, VT, USA). The results are expressed as μmol MDA equivalents mL/serum).

### 2.5. Statistical Analysis

The results were expressed as mean values ± standard error (SE) and were analyzed using the GraphPad software, version 6.0 (San Diego, CA, USA). All data were submitted to the normality test. Water consumption data were analyzed by Student T-test analysis. Other comparisons were performed by one-way ANOVA of variance with Tukey post-test and Kruskal-Wallis test with Dunn’s post-test. The statistical differences were represented by letters; the same letters located on top of the bars correspond to the absence of statistical difference, whereas different letters represent statistical difference among the groups. *p* < 0.05 was taken as indicative of statistical significance.

## 3. Results

### 3.1. Chemical Analysis

DPPH analysis (% scavenge) shows a higher percentage of radical sequestration with statistical difference between aqueous (39.9 ± 1.9) and methanol/acetone (27.6 ± 2.0) (*p* = 0.0061) and no difference with ethanol extract (34.2 ± 1.5). However, in the FRAP assays (μM FeSO_3_/ g sample), the aqueous extract presented a higher antioxidant activity (95.0 ± 5.9) compared with ethanol (64.2 ± 1.9) (*p* = 0.0017), but similar to that of methanol/acetone (80.0 ± 1.9). Using the ABTS assays (μM trolox/g sample), the aqueous extract demonstrated a higher antioxidant activity (35,792 ± 1044), followed by methanol/acetone (28,960 ± 760) and ethanol extract (24,775 ± 612), with statistical difference between the aqueous and ethanol extracts (*p* = 0.0012). The ORAC assays (μM trolox/g sample) did not demonstrate any statistical difference between the aqueous (1118 ± 83), ethanol (1021 ± 1) and methanol/acetone (1001 ± 119) extracts. The total phenolic compound analysis (mg GAE/g sample) showed a higher concentration in the aqueous extract (73.3 ± 1.0), compared with the ethanol extract (44.8 ± 0.8) (*p* = 0.0005), but no difference from the methanol/acetone extract was seen (64.8 ± 1.1).

Hydrolyzed tannins were identified in the three extracts of *E. uniflora* leaves using the qualitative method. The quantitative method (mg tannic acid/g sample) showed that ethanol extracted greater amounts of hydrolysable tannins (167.7 ± 1.5), in comparison with the aqueous (154.5 ± 3.2) and methanol/acetone (154.1 ± 4.2) extracts. There was a statistical difference only between the ethanol and methanol/acetone extracts (*p* = 0.0196).

### 3.2. Chromatographic Characterization (HPLC)

**Figure 2:**
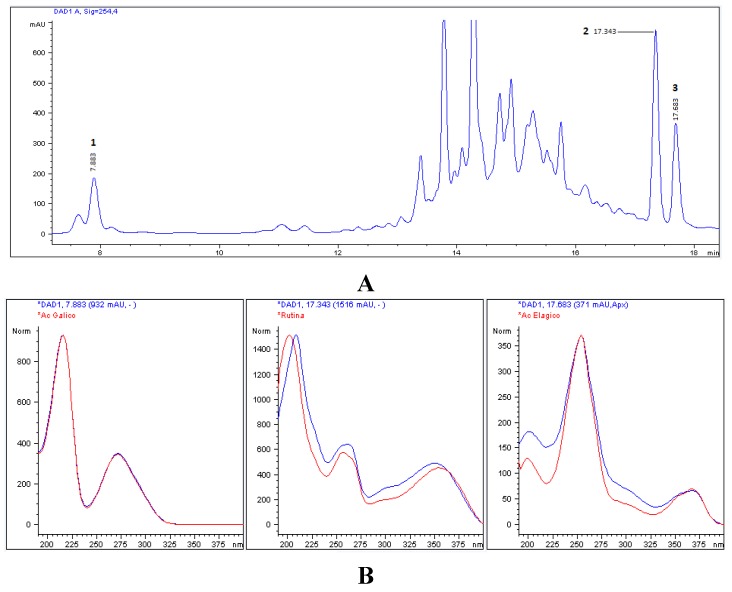
(**A**) Typical chromatogram of aqueous extract of *E. uniflora* leaves by HPLC-DAD (254 nm). Compounds: (1) gallic acid; (2) rutin and (3) ellagic acid. (**B**) Spectra of phenolic compounds identified in comparison with pure standards.

HPLC-DAD analysis using standard phenolic compounds (ellagic acid, gallic acid, vanillic acid, p-coumaric acid, ferulic acid, kaempferol, resveratrol, quercetin, catechin, epicatechin and rutin) was capable of identifying only ellagic acid, gallic acid and rutin in the extracts. However, the comparison of the contents of the extracts did not reveal any difference in the aqueous extract, as shown in Table 1. The phenolic compound peaks identified in the aqueous extract are shown in Figure 2A, where gallic acid and rutin were identified at 210 nm and ellagic acid was observed at 254 nm. Figure 2B shows the spectra of the identified compounds, comparing to the phenolic standard, with gallic acid and rutin at RTs of 7.883 min and 17.343 min, respectively, and ellagic acid at an RT of 17.683 min.

**Table 1:** Determination of phenolic compounds ellagic acid, galic acid and rutin of *E. uniflora* leaves by HPLC in aqueous, ethanol and methanol/acetone extracts.

Phenolic Compounds	Aqueous	Ethanol	Methanol/Acetone
Gallic acid	6402 ± 222.7 ^a,b^	7361 ± 115.7 ^a^	2677 ± 32.3 ^b^
Rutin	10026 ± 97.2 ^b^	18542 ± 142.6 ^a,b^	20210 ± 25.2 ^a^
Ellagic acid	2462 ± 55.4 ^b^	4869±48.8 ^a^	3389 ± 40.5 ^a,b^

Results are expressed as means ± SE (mg/L) of triplicate experiments. Different letters show significant differences among the extracts. Kruskal-Wallis test with Dunn’s post-test; statistical markers ^a/b^: *p* < 0.05.

### 3.3. Biological Assays: NOD Mice Treated with E. uniflora Leaves

#### 3.3.1. Consumption of Water and Aqueous Extract of *E. uniflora* and Cumulative Frequency of Incidence of DM-1

Aqueous extract was chosen for biological assay and *in vivo* treatment response of spontaneous type 1 diabetes NOD mice due to its higher antioxidant activity when compared with ethanol and methanol/acetone.

The results of the daily water ingestion of untreated group (4.7 ± 0.7 mL/animal) and aqueous extract ingestion of treated group (5.7 ± 1.1 mL/animal) did not show significant differences. The crude extract equivalence intake was 3.3 mg/daily/animal. Moreover, from HPLC analysis, we calculated the relative daily intake/animal of gallic acid, rutin and ellagic acid present in the extract, *i.e.*, 0.5 mg, 0.8 mg and 0.2 mg, respectively.

**Figure 3:**
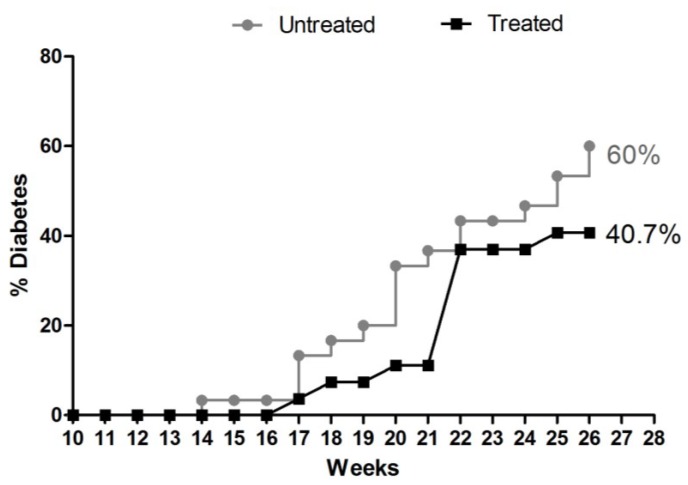
Cumulative frequency of incidence of DM-1 in NOD mice subjected to treatment with *E. uniflora* aqueous extract or in untreated mice. Sixty percent (18/30) of the untreated group developed diabetes compared with 40.7% (11/27) in the group treated with aqueous extract of *E. uniflora*, showing a difference of 19.3% between frequencies.

The treatment of NOD mice with aqueous extract of *E. uniflora* reduced the manifestations of diabetes. Thus, 40.7% of the treated animals (11/27) became diabetic in contrast to 60% of untreated animals (18/30), with a difference of 19.3% between frequencies (Figure 3).

#### 3.3.2. Effect of Aqueous Extract of *E. uniflora* on Inflammation and Infiltration Index in Islet

**Figure 4:**
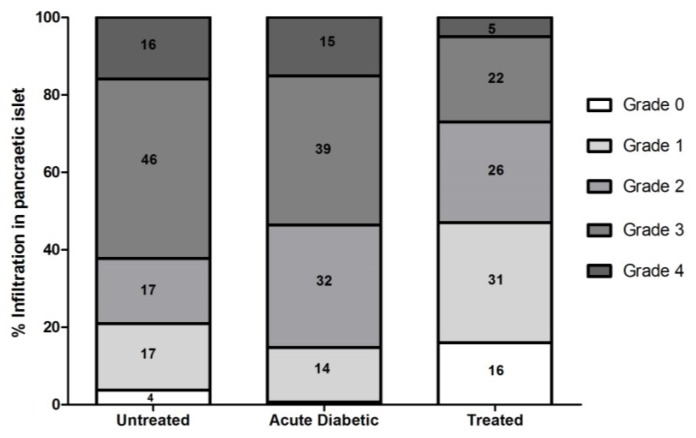
Classification of pancreatic islets, according to the degree of insulitis in untreated NOD (*n* = 5), treated NOD (*n* = 5) and acute diabetic NOD (*n* = 5) mice. Total number of islets examined in each group = 300. Each group represents 100%, where the diabetic group showed only 1% grade 0 islets.

Morphological analysis by HE staining, followed by insulitis classification, revealed that animals treated with *E. uniflora* aqueous extract have more grade 1 and 2 islets compared with the untreated group and acute diabetic group, which had a higher number of grade 2 and 3 islets. More grade 0 insulitis was observed in the treated (*n* = 16), followed by untreated (*n* = 4) and acute diabetic (*n* = 1) groups. We also observed large amounts of insulitis grade 4 in untreated (*n* = 16) and acute diabetic (*n* = 15) groups, compared with the treated group (*n* = 5) (Figure 4). From the insulitis index, treated animals showed a lower insulitis index (0.43 ± 0.048) than untreated (0.63 ± 0.05) and acute diabetic groups (0.65 ± 0.03) (*p* = 0.0137).

#### 3.3.3. Effects of Aqueous Extract on Serum Insulin Concentration

**Figure 5:**
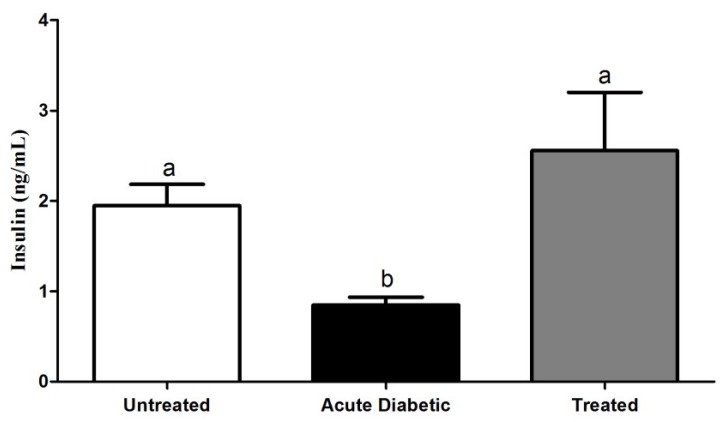
Serum insulin concentration, as determined by ELISA, in NOD mice in untreated (*n* = 15), acute diabetic (*n* = 15) and treated (*n* = 15) groups. Results are expressed as mean ± SE; the different letters represent statistical difference. Kruskal–Wallis test with Dunn’s post-test. Statistical markers a/b: *p* < 0.05.

The insulin serum concentration was significantly decreased in the diabetic group (0.85 ± 0.09 ng/mL) (*p* < 0.05) and there was no difference between untreated (1.95 ± 0.2 ng/mL) and treated (2.56 ± 0.6 ng/mL) groups (Figure 5). The damage of insulin-producing beta cells that occur in type 1 diabetes decreases insulin availability, leading to hyperglycemic state. Our results suggest that components of *Eugenia* extract favourably modulate the insulin/glucose process, decreasing the glucose intolerance.

#### 3.3.4. Glucose Tolerance Test in NOD and BALB/c Mice

**Figure 6:**
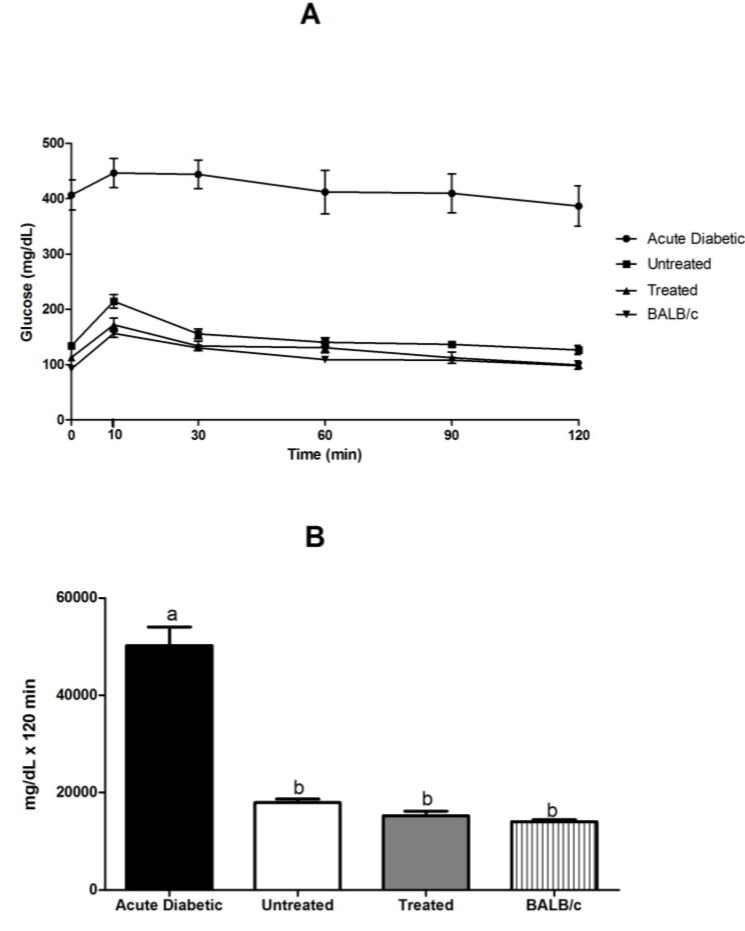
Blood glucose (mg/dL) (**A**) and area under the glucose curve (**B**) in NOD mice related to diabetic, untreated, treated and BALB/c mice groups. Results are expressed as mean ± SE; the different letters represent statistical difference. Statistical test of Kruskal–Wallis test with Dunn’s post-test, considering the diabetic group as a reference to analysis. Statistical markers a/b: *p* < 0.05.

GTT is an auxiliary method for diagnosing diabetes mellitus or insulin resistance. Our results showed that blood glucose (mg/dL) versus time (min) in the group treated with *E. uniflora* leaves resembled that of the group BALB/c, signifying that treatment improved insulin release and tolerance (Figure 6A). Thus, we found statistical difference (Figure 6B) in the analysis of the area under the curve (AUC) of blood glucose for the acute diabetic group (48,074 ± 4882), compared with the other groups of untreated (18,240 ± 851.4) (*p* < 0.05), treated (15,206 ± 1259) (*p* < 0.001) and BALB/c (13,241 ± 4488) (*p* < 0.0001) mice.

#### 3.3.5. Reduced Glutathione Analysis

**Figure 7:**
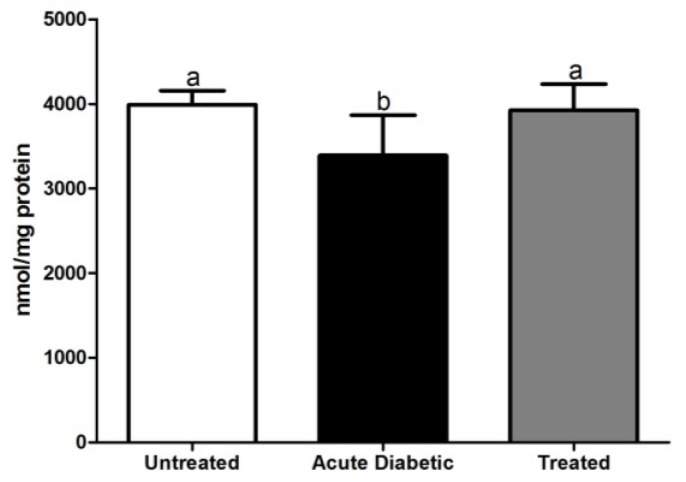
Concentration of glutathione present in the liver of untreated, diabetic and treated NOD mice. Results are expressed as mean ± SE; the different letters represent statistical difference between the groups. Statistical test of Kruskal-Wallis test with Dunn’s post-test. Statistical markers a/b: *p* < 0.05.

Hepatic glutathione in untreated non-diabetic animals (3992 ± 58.5 nmol/mg) and in the treated group (3926 ± 109 nmol/mg) showed higher glutathione levels (*p* < 0.05) than the diabetic group (3390 ± 119 nmol/mg) (Figure 7).

#### 3.3.6. Determination of Serum Antioxidant Activity and Lipid Peroxidation Assay

**Figure 8:**
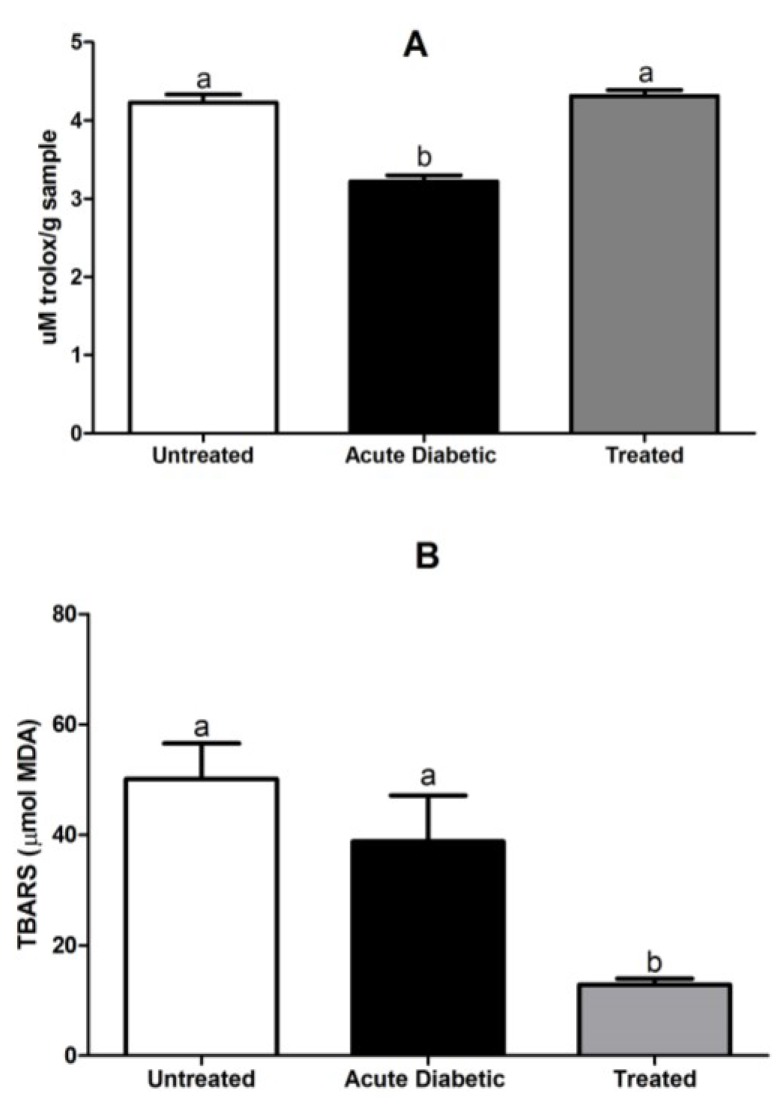
(**A**) Antioxidant activity of ABTS in serum of untreated, acute diabetic and treated NOD mice (a/b: *p* < 0.001). (**B**) Concentration of TBARS in the serum of untreated, acute diabetic and treated NOD mice (a/b: *p* < 0.01). Results are expressed as mean ± SE; the different letters represent statistical difference between the groups. Statistical test of Kruskal-Wallis test with Dunn’s post-test.

Serum antioxidant activity in the ABTS assays was significantly decreased in the acute diabetic group (3.1 ± 0.08) (*p* < 0.001) and there was no difference between treated (4.3 ± 0.07) and untreated (4.2 ± 0.11) animals (Figure 8A).

The animals treated with aqueous extracts of *E. uniflora* leaves (12.8 ± 1.1) (*p* < 0.01) significantly decreased lipid peroxidation compared to the untreated (50.1 ± 6.4) and acute diabetic (38.8 ± 8.3) animals (Figure 8B).

## 4. Discussion

Phenolic compounds present in foods (fruits and vegetables) may prevent or control some diseases due to their antioxidant, anti-inflammatory, anticancer and antimicrobial properties [31,32].

In this study, we evaluated the extraction efficiency of three different solvents (aqueous, ethanol and methanol/acetone), considering the amounts of phenolic compounds in dried of *E. uniflora* leaves. Although solvent ethanol and methanol are used and very effective for the extraction of phenolic compounds [33], our results demonstrated that boiling water (autoclaving) is the best extraction solvent. Our previous study reported that *Passilfora alata* leaves (sweet passion fruit), when subjected to aqueous extraction at high temperature, yields components with anti-inflammatory properties that reduce the infiltration of inflammatory cells in pancreatic islets, consequently reducing the incidence of diabetes in NOD mice [9].

The antioxidant activity of the extracts was evaluated by four methods (DPPH, FRAP, ABTS and ORAC), where differences in results may have occurred due to the interaction of the solvents used, chemical structure, pH and solvent polarity. The presence of non-antioxidant food components (amino acids and uronic acids) may interfere in the quantification of antioxidant activity in food [34]. Pérez-Jiménez and Calixto (2006) [34], studying the antioxidant capacity of catechin and gallic acid, using DPPH, ABTS, FRAP and ORAC with different solvents (water, methanol/water, methanol, acetone/water), found differences among the methods. ORAC showed a higher affinity for a nonpolar solvent, in contrast to ABTS with affinity for the polar solvent. However, the DPPH and FRAP assays did not demonstrate any interference due to solvent polarity. Bagetti *et al*. (2011) [35] quantified the antioxidant activity of three *E. uniflora* pulps (purple, red and orange), which showed high antioxidant activity for the DPPH and FRAP methods, as demonstrated for the leaf extracts.

Total phenols have antioxidant activity, acting in the neutralization of free radicals, and contributing to the control of oxidative stress in pancreatic islets of diabetic mice [36]. Our results regarding the quantification of total phenols show that aqueous extract of *E. uniflora* has strong antioxidant activity suggesting anti-inflammatory properties, as demonstrated by the *in vivo* model studies group [9].

Some factors, such as location, seasonality and the collection season, can influence the concentration of bioactive compounds present in plants and the biosynthesis of secondary metabolites (flavonoids), influencing their antioxidant potential and their biological effects [37,38].

Santos *et al.* (2011) [39] studied phenolic compounds from *E. uniflora* leaves at different times of the year; the leaves were dried, ground and, after using acetone as the solvent extractor, the material was lyophilized. Our results, regarding total phenolic content and hydrolysable tannins, were similar for the leaves collected in September, *i.e*., 82.61 mg/g and 112.21 mg/g samples, respectively.

Tannins have antioxidant and anti-inflammatory properties, and for the three extracts tested (aqueous, ethanol and methanol/acetone), the presence of hydrolysable tannins was detected (gallic acid and ellagic acid). Some studies have reported the presence of hydrolyzed tannins in ethanolic and methanolic extracts of *E. uniflora* leaves, but they did not assess the aqueous extract [18,40].

Component analysis of the three extracts by HPLC-DAD chromatography showed the presence of three phenolic compounds (gallic acid, ellagic acid and rutin). Oliveira *et al*. (2014) [41] analyzing seeds of *E. uniflora,* identified the presence of ellagic acid, quercetin and kaempferol in the ethanol extract.

Al-Salih (2010) [42], studying the synergistic action of two hydrolysable tannins (tannic and gallic acids), found that the mixed components had a higher antioxidant activity and antidiabetic action than the isolated compounds. Kamalakkannan and Stanley (2006) [43], studying the action of rutin in diabetic rats verified a decrease in the oxidative stress (liver, kidney and brain), supporting the hypothesis of pancreatic β cell protection, *i.e*., improvement in insulin secretion and decrease in hyperglycemia. The study of the properties of antioxidant phenolic compounds in the diseases related to oxidative stress (such as diabetes, obesity, cardiovascular disease, metabolic syndrome, neurodegenerative diseases, cancer, among others) is attractive due to the growing popularity of herbal medicines, resulting from their bioactive characteristics that help prevent these diseases [44]. The development of diabetes is the result of β-cell destruction (insulin-production) in the pancreatic islets by an inflammatory process that can be generated by oxidative stress. Colomeu *et al.* (2014) [9] showed that NOD mice treated with antioxidants (present in the aqueous extract of *Passiflora alata* leaves) showed a decrease in the oxidative stress of pancreatic islets.

NOD mice were submitted to chronic treatment (26 weeks) with aqueous extract of *E. uniflora* leaves (the best antioxidant extract). It was found difference of 19.3% in diabetes expression. This difference can be represented by subpopulations more susceptible to treatment, which could be compared with the different genotypes in response to treatment of human disease. However, the possibility of dose-dependent effects on diabetes is not ruled out.

Insulitis in NOD mice starts at the 4th week of life with progression of infiltrating inflammatory cells, which occurs in parallel with β-cell destruction [45]. As such, insulitis significantly decreased in the treated group, possibly due to the presence of phenolic compounds in the leaves of *E. uniflora.*

The highest concentration of insulin in the treated group support the hypothesis of a reduction in the inflammatory infiltrate, since the effect of the aqueous extract may be related to the preservation of the pancreatic islet and, therefore, insulin-producing β cells.

The glucose tolerance test can be used to determine whether mice submitted to high concentrations of glucose are diabetic. Parameters that may interfere with the glucose tolerance test include the fasting time, glucose dose and route of administration and mice consciousness [26]. Thus, these properties could have altered the glucose tolerance test in these mice, causing blood glucose levels to be lower in the treated group than in the acute diabetic group.

Antioxidant defenses can be classified as endogenous (GSH, SOD and CAT) and exogenous (dietary) that can act direct or indirectly on many biological processes including protein synthesis, metabolism and cell protection [4,46]. Kalkan and Suher (2013) [47] analyzed GTT in three groups of patients by fasting glucose analysis: diabetics, individuals with impaired glucose tolerance (IGT) and normal patients. They concluded that there is a reduction in GSH levels in diabetic patients compared with IGT patients. Sekhar *et al.* (2011) [48] studying human type 2 diabetes with dietary supplementation of cysteine and glycine (precursor amino acids) verified high levels of intracellular GSH and the reduction of oxidative damage markers and oxidative stress in these patients. These findings enforce our GSH results, showing that chronic treatment using *E. uniflora* aqueous extract increases hepatic GSH levels when compared with acute diabetic animals.

TBARS is a marker of oxidative stress, formed from the degradation of oxidation products of unsaturated fatty acids by ROS, and plays an important role in complications of diabetes [49]. Dave and Kalia (2007) [50] showed high levels of TBARS in plasma from diabetic patients compared to the control group [51]. Nakhaee *et al.* [52] also showed a significant increase in TBARS in the plasma and liver of diabetic rats, compared to the control group. Jelenik *et al.* (2014) [53] studying the lipid peroxidation in liver and muscle of NOD mice verified a high level of ROS associated to oxidative stress in the liver of chronic diabetes. However, in contrast to these findings the level of ROS associated with oxidative stress in the muscle was not different for the non-diabetic, acute diabetic and chronic diabetic models. Thus, these data are in agreement with our results obtained by TBARS, when comparing untreated with acute diabetic mice, reinforcing the ability of chronic treatment with aqueous extract *Eugenia uniflora* to reduce lipid peroxidation in NOD mice. Serum antioxidant activity (ABTS) were higher in the treated and untreated groups, compared to the acute diabetic group. As such, the acute diabetic animals showed a greater imbalance in serum antioxidant activity and increased concentrations of oxidative stress markers [54].

## 5. Conclusions

The analysis of antioxidant activity among aqueous, ethanol and methanol-acetone extracts show that the aqueous extract has a higher antioxidant activity. It is possible that the presence of major compounds found in these three extracts (ellagic acid, gallic acid and rutin) contribute to the anti-inflammatory and antidiabetic effects of the extracts.

This is the first report to look at the effects of chronic treatment of aqueous extract of dried leaves of *E. uniflora* on the expression of markers of diabetes in NOD mice. The treatment reduced the incidence of DM-1, decreased inflammatory cell infiltration and the oxidative stress, and increased hepatic glutathione levels and serum insulin, which may suggest preservation of insulin-producing pancreatic β cells. Overall, results are very promising and may demonstrate the action of phenolic compounds present in *E. uniflora* leaves, which have antioxidant and anti-inflammatory properties. However, despite the anti-inflammatory properties found during this study, further studies are necessary to determine the dose/response of aqueous extract and the anti-inflammatory signalling process of immune response in pancreas islets, aiming to consolidate these results and justify clinical trial development.
